# Supplementary material for: The Relationship between Coronary Artery Wall Shear Strain and Plaque Morphology: A Systematic Review and Meta-Analysis
Source: Diagnostics (Basel). 2020 Feb 8;10(2):91. doi: 10.3390/diagnostics10020091 (PMC7168174; doi:10.3390/diagnostics10020091)

Supplement material

Supplement 1. Qualitative assessment of study reporting.

Domain	Questions	Judgments
**Risk of bias**		
1) Patient selection	*Was a consecutive or random sample of patients enrolled?*	Yes, No, Unclear
	Was a case–control design avoided?	Yes, No, Unclear
	Did the study avoid inappropriate exclusions?	Yes, No, Unclear
	Could the selection of patients have introduced bias?	Low, High, Unclear
**Applicability** 1) Patient selection	*Is there concern that the included patients do not match the review questions?*	Low, High, Unclear
**Risk of bias** 2) Index test	*Were the index test results interpreted without knowledge of the results of the reference standard?*	Yes, No, Unclear
	If a threshold was used, was it pre-specified?	Yes, No, Unclear
	Could the conduct or interpretation of the index test have introduced bias?	Low, High, Unclear
**Applicability** 2) Index test	*Is there concern that the index test, its conduct, or interpretation differ from the review question?*	Low, High, Unclear
**Risk of bias** 3) Reference standard	*Is the reference standard likely to correctly classify the target condition?*	Yes, No, Unclear
	Were the reference standard results interpreted?	Yes, No, Unclear
	If Yes, was it without knowledge of the results of the index test?	Yes, No, Unclear
	Could the reference standard, its conduct, or its interpretation have introduced bias?	Low, High, Unclear
**Applicability** 3) Reference standard	*Is there concern that the target condition as defined by the reference standard does not match the review question?*	Low, High, Unclear
**Risk of bias** 4) Flow and timing	*Was there an appropriate interval between index test(s) and reference standard?*	Yes, No, Unclear
	Did all patients receive a reference standard?	Yes, No, Unclear
	Did patients receive the same reference standard?	Yes, No, Unclear
	Were all patients included in the analysis?	Yes, No, Unclear
	Could the patient flow have introduced bias?	Low, High, Unclear

Patients: Patients with CAD; different types of WSS.

Index test: Relationship between types of WSS and plaque morphology.

Comparator test (if applicable): Baseline features of vulnerability of coronary artery in different types of WSS.

Target condition: Role of types of WSS in coronary plaque; reference standard: morphology of coronary plaque.

Supplementary 2. Comparison of baseline lumen area, plaque area, plaque burden, and necrotic core in group of intermediate WSS vs. high WSS.

a) Baseline lumen area

Study or Subgroup	High WSS Mean	High WSS SD	High WSS Total	Intermediate WSS Mean	Intermediate WSS SD	Intermediate WSS Total	Weight	Mean Difference IV, Random, 95% CI	Year
Samady 2011	8.2	3.8	1010	11.4	4.1	1034	33.5%	-3.20 [-3.54, -2.86]	2011
Eshtehardi 2012	7.5	6.2	3851	8.9	7.9	3851	33.6%	-1.40 [-1.72, -1.08]	2012
Han 2016	2.5	1.4	55	2.7	1.4	54	32.9%	-0.20 [-0.73, 0.33]	2016
**Total (95% CI)**			**4916**			**4939**	**100.0%**	**-1.61 [-3.21, -0.01]**	

Heterogeneity: Tau² = 1.95; Chi² = 10.03, df = 2 (P = 0.01); I² = 68%
Test for overall effect: Z = 1.97 (P = 0.05)

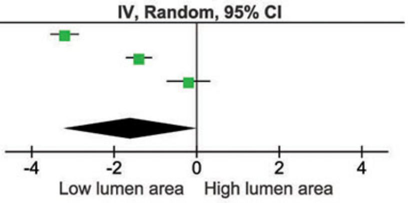

b) Baseline plaque area

Study or Subgroup	High WSS Mean	High WSS SD	High WSS Total	Intermediate WSS Mean	Intermediate WSS SD	Intermediate WSS Total	Weight	Mean Difference IV, Random, 95% CI	Year
Samady 2011	7.1	3.7	1010	6.1	3.1	1034	32.5%	1.00 [0.70, 1.30]	2011
Eshtehardi 2012	5.8	4.8	3851	4.8	3.6	3851	33.4%	1.00 [0.81, 1.19]	2012
Timmins 2017	0.45	0.4	5964	0.45	0.56	5964	34.0%	0.00 [-0.02, 0.02]	2017
**Total (95% CI)**			**10825**			**10849**	**100.0%**	**0.66 [-0.14, 1.46]**	

Heterogeneity: Tau² = 0.49; Chi² = 14.96, df = 2 (P = 0.007); I² = 59%
Test for overall effect: Z = 1.61 (P = 0.11)

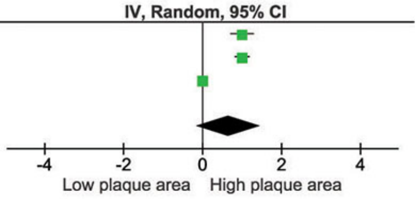

c) Baseline plaque burden

Study or Subgroup	High WSS Mean	High WSS SD	High WSS Total	Intermediate WSS Mean	Intermediate WSS SD	Intermediate WSS Total	Weight	Mean Difference IV, Random, 95% CI	Year
Samady 2011	45.5	5.9	1010	34.3	12.4	1034	51.6%	11.20 [10.36, 12.04]	2011
Eshtehardi 2012	39.4	34	3851	31.4	28	3851	48.4%	8.00 [6.61, 9.39]	2012
**Total (95% CI)**			**4861**			**4885**	**100.0%**	**9.65 [6.52, 12.78]**	

Heterogeneity: Tau² = 4.78; Chi² = 14.91, df = 1 (P = 0.0001); I² = 53%
Test for overall effect: Z = 6.03 (P < 0.00001)

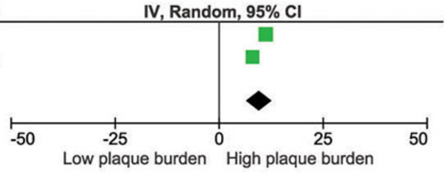

d) Baseline necrotic core

Study or Subgroup	High WSS Mean	High WSS SD	High WSS Total	Intermediate WSS Mean	Intermediate WSS SD	Intermediate WSS Total	Weight	Mean Difference IV, Random, 95% CI
Samady 2011	0.8	0.8	1010	0.56	0.7	1034	48.6%	0.24 [0.17, 0.31]
Timmins 2017	0.13	0.2	3127	0.09	0.16	5963	51.4%	0.04 [0.03, 0.05]
**Total (95% CI)**			**4137**			**6997**	**100.0%**	**0.14 [-0.06, 0.33]**

Heterogeneity: Tau² = 0.02; Chi² = 35.57, df = 1 (P < 0.00001); I² = 67%
Test for overall effect: Z = 1.37 (P = 0.17)

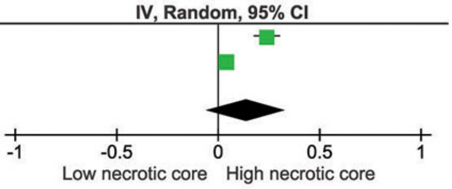

Supplement 3. Comparison of baseline dense calcium, fibrous, and fibro-fatty area in group of intermediate WSS vs. high WSS.

a) Baseline dense calcium

Study or Subgroup	High WSS Mean	High WSS SD	High WSS Total	Intermediate WSS Mean	Intermediate WSS SD	Intermediate WSS Total	Weight	Mean Difference IV, Random, 95% CI
Samady 2011	0.37	0.53	1010	0.24	0.42	1034	48.9%	0.13 [0.09, 0.17]
Timmins 2017	0.05	0.05	3127	0.06	0.15	5694	51.1%	-0.01 [-0.01, -0.01]
**Total (95% CI)**			**4137**			**6728**	**100.0%**	**0.06 [-0.08, 0.20]**

Heterogeneity: Tau² = 0.05; Chi² = 3.22, df = 1 (P = 0.05); I² = 48%
Test for overall effect: Z = 0.83 (P = 0.40)

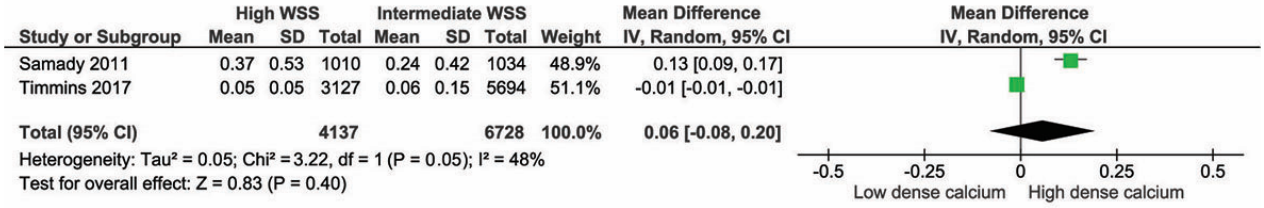

b) Baseline fibrous area

Study or Subgroup	High WSS Mean	High WSS SD	High WSS Total	Intermediate WSS Mean	Intermediate WSS SD	Intermediate WSS Total	Weight	Mean Difference IV, Random, 95% CI
Samady 2011	2.5	2.05	1010	1.78	1.66	1034	48.9%	0.72 [0.56, 0.88]
Timmins 2017	0.41	0.42	5144	0.25	0.33	5964	51.1%	0.16 [0.15, 0.17]
**Total (95% CI)**			**6154**			**6998**	**100.0%**	**0.43 [-0.11, 0.98]**

Heterogeneity: Tau² = 0.15; Chi² = 5.59, df = 1 (P = 0.02); I² = 38%
Test for overall effect: Z = 1.55 (P = 0.12)

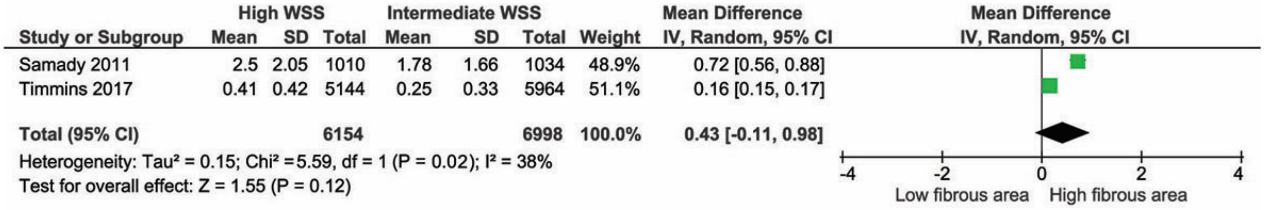

c) Baseline fibrofatty area

Study or Subgroup	High WSS Mean	High WSS SD	High WSS Total	Intermediate WSS Mean	Intermediate WSS SD	Intermediate WSS Total	Weight	Mean Difference IV, Random, 95% CI
Samady 2011	0.51	0.61	1010	0.26	0.33	1011	49.6%	0.25 [0.21, 0.29]
Timmins 2017	0.07	0.13	3127	0.05	0.13	5694	50.4%	0.02 [0.01, 0.03]
**Total (95% CI)**			**4137**			**6705**	**100.0%**	**0.13 [-0.09, 0.36]**

Heterogeneity: Tau² = 0.07; Chi² = 10.18, df = 1 (P = 0.08); I² = 39%
Test for overall effect: Z = 1.17 (P = 0.24)

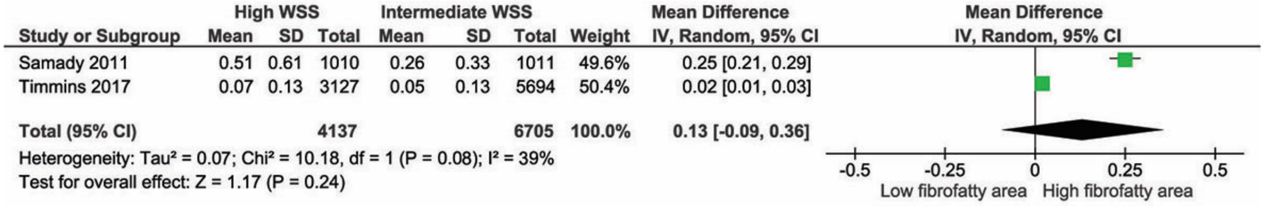

Supplementary 4. Comparison of baseline lumen area, plaque area, plaque burden, and necrotic core in group of low WSS vs. intermediate WSS.

a) Baseline lumen area

	Low WSS			Intermediate WSS				Mean Difference	
Study or Subgroup	Mean	SD	Total	Mean	SD	Total	Weight	IV, Random, 95% CI	Year
Samady 2011	11.4	4.5	205	11.4	4.1	1034	32.4%	0.00 [-0.66, 0.66]	2011
Eshtehardi 2012	10.7	9.9	3851	8.9	7.9	3851	35.2%	1.80 [1.40, 2.20]	2012
Han 2016	3.7	2.1	55	2.7	1.4	54	32.4%	1.00 [0.33, 1.67]	2016
**Total (95% CI)**			**4111**			**4939**	**100.0%**	**0.96 [-0.12, 2.04]**	

Heterogeneity: Tau² = 0.82; Chi² = 21.38, df = 2 (P = 0.01); I² = 68%
Test for overall effect: Z = 1.74 (P = 0.08)

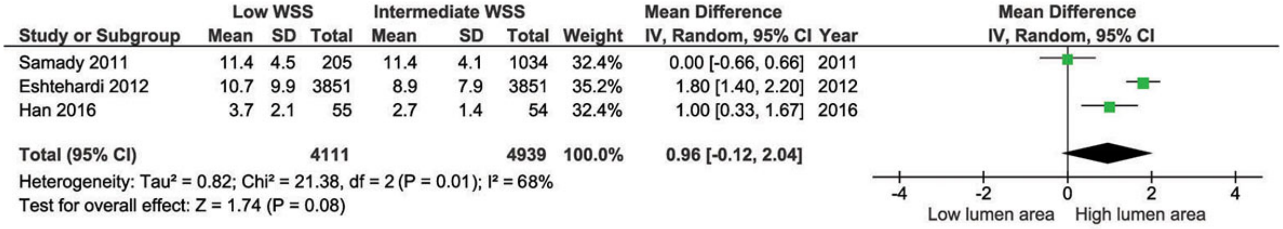

b) Baseline plaque area

	Low WSS			Intermediate WSS				Mean Difference	
Study or Subgroup	Mean	SD	Total	Mean	SD	Total	Weight	IV, Random, 95% CI	Year
Samady 2011	4.6	2.1	205	6.1	3.1	1034	30.9%	-1.50 [-1.84, -1.16]	2011
Eshtehardi 2012	5.1	3.2	3851	4.8	3.3	3851	34.2%	0.30 [0.15, 0.45]	2012
Timmins 2017	0.25	0.4	3127	0.45	0.56	5964	35.0%	-0.20 [-0.22, -0.18]	2017
**Total (95% CI)**			**7183**			**10849**	**100.0%**	**-0.43 [-0.99, 0.13]**	

Heterogeneity: Tau² = 0.23; Chi² = 80.18, df = 2 (P < 0.00001); I² = 78%
Test for overall effect: Z = 1.51 (P = 0.13)

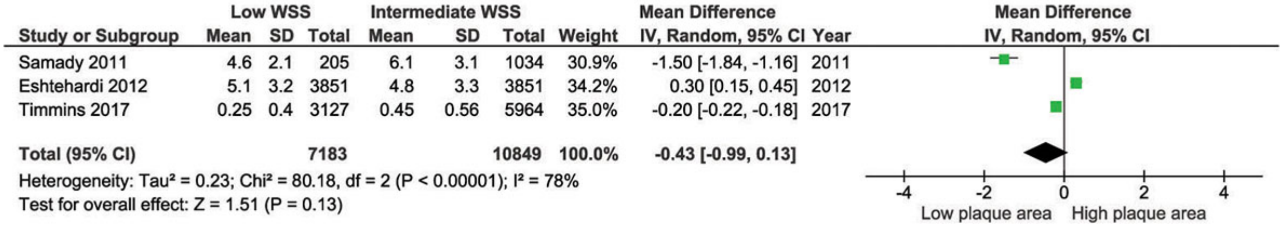

c) Baseline plaque burden

	Low WSS			Intermediate WSS				Mean Difference	
Study or Subgroup	Mean	SD	Total	Mean	SD	Total	Weight	IV, Random, 95% CI	Year
Samady 2011	28.3	10.3	205	34.3	12.4	1034	49.6%	-6.00 [-7.60, -4.40]	2011
Eshtehardi 2012	31.1	27	3851	31.4	28	3851	50.4%	-0.30 [-1.53, 0.93]	2012
**Total (95% CI)**			**4056**			**4885**	**100.0%**	**-3.13 [-8.71, 2.46]**	

Heterogeneity: Tau² = 5.72; Chi² = 10.68, df = 1 (P = 0.08); I² = 57%
Test for overall effect: Z = 1.10 (P = 0.27)

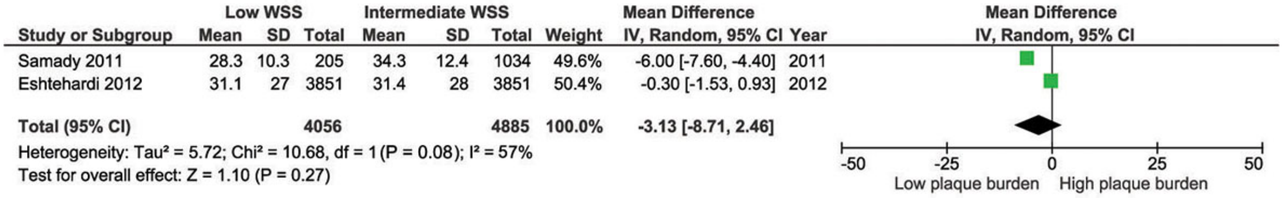

d) Baseline necrotic core

	Low WSS			High WSS				Mean Difference
Study or Subgroup	Mean	SD	Total	Mean	SD	Total	Weight	IV, Random, 95% CI
Samady 2011	0.24	0.39	205	0.56	0.7	1034	49.2%	-0.32 [-0.39, -0.25]
Timmins 2017	0.04	0.1	3127	0.09	0.16	5963	50.8%	-0.05 [-0.06, -0.04]
**Total (95% CI)**			**3332**			**6997**	**100.0%**	**-0.18 [-0.45, 0.08]**

Heterogeneity: Tau² = 0.04; Chi² = 9.59, df = 1 (P = 0.01); I² = 58%
Test for overall effect: Z = 1.35 (P = 0.18)

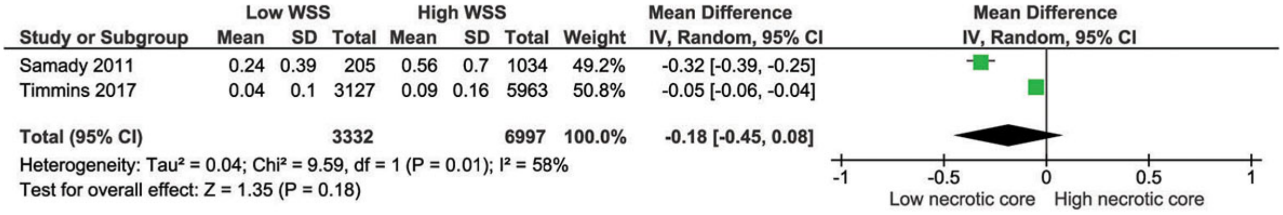

Supplementary 5. Comparison of baseline dense calcium, fibrous, and fibro fatty area in group of low WSS vs. intermediate WSS.

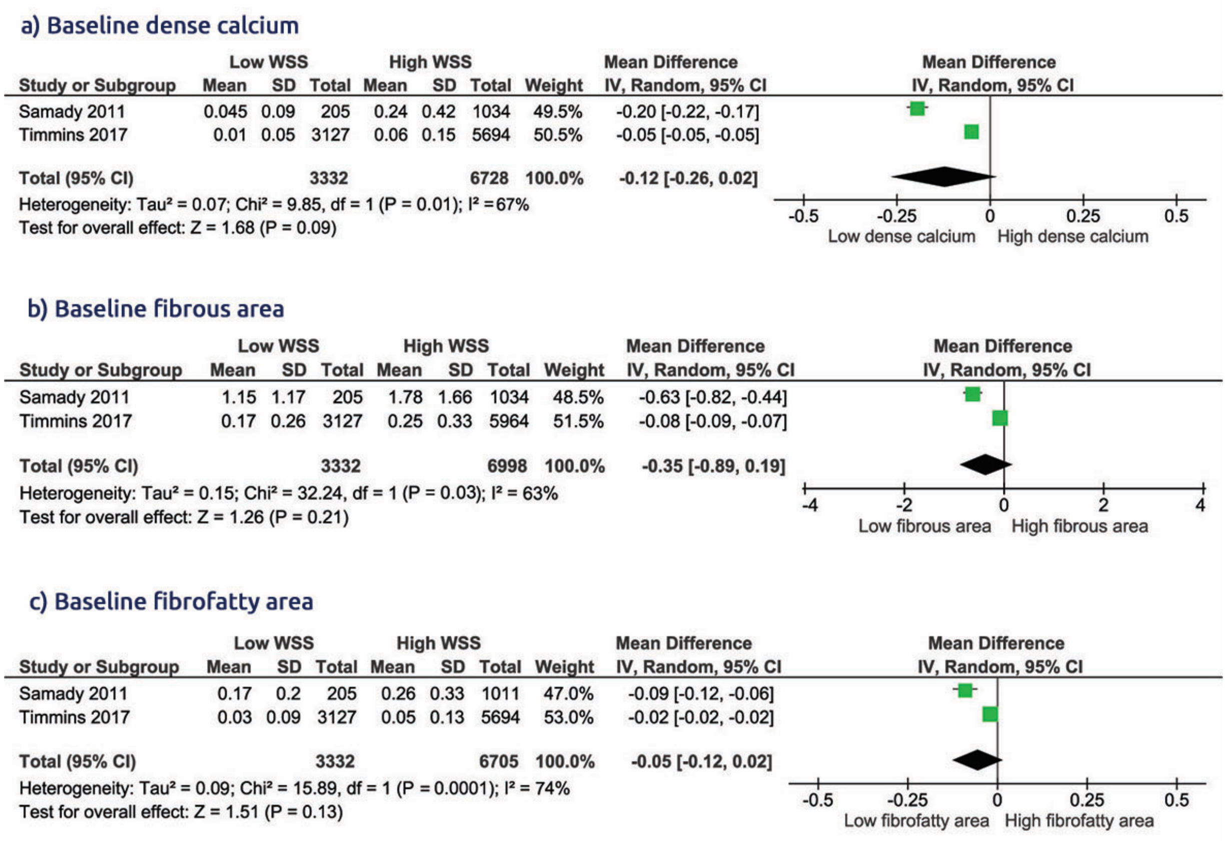

Supplementary 6. Summary of QUADAS-2 assessment of selected studies.

**Author**	**Risk of bias**				**Applicability concerns**		
**(year)**	**Patients selection**	**Index test**	**Reference standard**	**Flow and timing**	**Patients selection**	**Index test**	**Reference standard**
Samady 2011	Low	Low	Low	Low	Low	Low	Low
Eshtehardi 2012	Unclear	Low	Unclear	Low	Unclear	Unclear	Unclear
Timmins 2015	Low	Unclear	Low	Low	Unclear	Unclear	Low
Timmins 2017	Low	Low	Low	Low	Low	Low	Low

QUADAS-2: Quality Assessment of Diagnostic Accuracy Studies-2.

Supplementary 7. Summary of quality assessment analysis (Quality Assessment of Diagnostic Accuracy Studies-QUADAS 2).

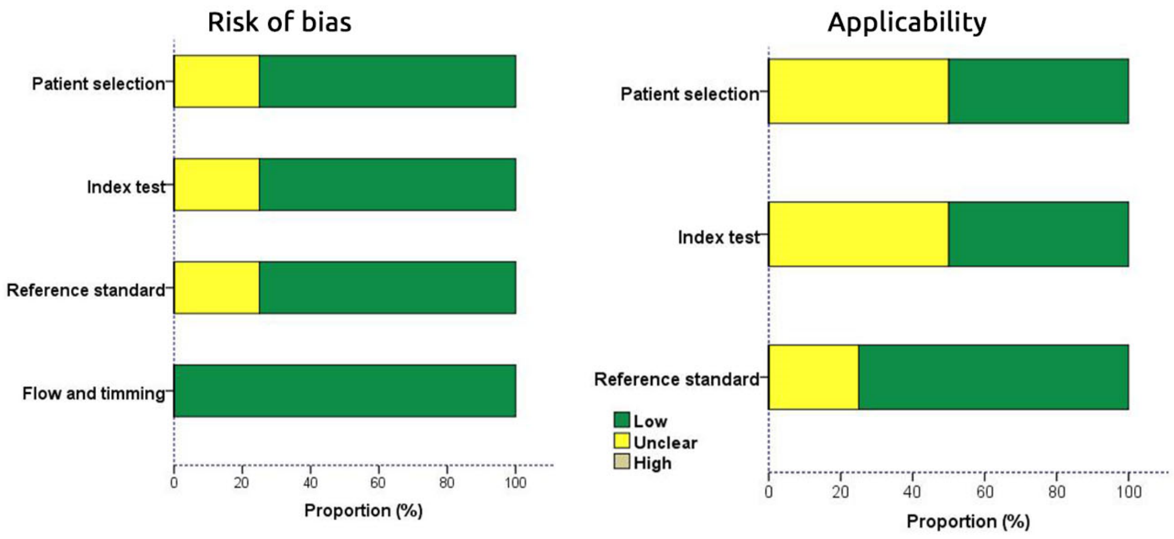

Supplement: Supplementary file 1 [file diagnostics-10-00091-s001.pdf]
